# Clinical practice guidelines on nutrition management in head and neck cancer: a systematic quality appraisal using the Appraisal of Guidelines for Research and Evaluation 2nd edition instrument

**DOI:** 10.1017/S002221512200055X

**Published:** 2023-01

**Authors:** K Panara, B Go, M Shah, T Majmudar, L-X Barrette, A G Moreira, K Rajasekaran

**Affiliations:** 1 Department of Otorhinolaryngology, University of Pennsylvania, Philadelphia; 2 Perelman School of Medicine, University of Pennsylvania, Philadelphia; 3 Leonard Davis Institute of Health Economics, University of Pennsylvania, Philadelphia; 4 College of Medicine, Drexel University, Philadelphia; 5 Department of Pediatrics, University of Texas Health Science Center at San Antonio, USA

**Keywords:** Head And Neck Neoplasms, Nutritional Support, Malnutrition, Guidelines, Consensus

## Abstract

**Objective:**

Several guidelines have been produced for the management of nutrition in patients with head and neck cancer. However, no systematic evaluation of the quality of these guidelines has been performed to date.

**Method:**

A comprehensive search was conducted up to August 2020. The quality of guidelines was assessed by four independent reviewers using the Appraisal of Guidelines for Research and Evaluation, 2nd edition.

**Results:**

Nine guidelines were assessed for critical evaluation. Only two guidelines were classified as ‘high quality’. The ‘scope and purpose’ domain achieved the highest mean score (75.5 ± 17.0 per cent), and the lowest domain mean score was ‘applicability’ (37.6 ± 23.0 per cent).

**Conclusion:**

These findings highlight the variability in the methodological quality of guidelines for the management of nutrition in head and neck cancer. These results may help to improve the reporting of future guidelines and guide the selection for use in clinical practice.

## Introduction

Head and neck cancers include malignancies that involve the epithelia of the upper digestive tract, encompassing the oral cavity, nasal cavity, paranasal sinuses, oropharynx, larynx and salivary glands.^1^ The epidemiology of head and neck cancer has changed significantly over the past few decades. Estimates indicate the incidence of late stage head and neck cancers to be 7.7 per 100 000 person-years, with the disease disproportionately affecting males and African Americans.^2^ More specifically, human papilloma virus associated cancer has increased dramatically, whereas the typical association of head and neck cancer with tobacco and alcohol use has slowly declined because of changing trends in substance use.^1^ Head and neck cancers account for approximately 4 per cent of cancer diagnoses in the USA annually and are responsible for a significant reduction in a patient's quality of life.^2^

Malnutrition is common in a variety of cancer types, but patients with head and neck cancer are particularly prone to malnutrition with up to 57 per cent of patients presenting with a more than 10 per cent weight loss from baseline body mass.^3,4^ Alcohol and tobacco use are traditionally implicated in the pre-treatment causes of malnutrition because of associated poor dietary intake of nutrients and calories. In addition, head and neck cancers can also cause anatomical and physiological disturbances in the gastrointestinal tract, including dysphagia, aspiration, trismus and odynophagia. Adequate nutrition in head and neck cancer is further complicated by treatment modalities that often cause functional impairments. For example, surgery can disrupt normal mechanisms of swallowing or cause gastrointestinal discontinuity. In addition, the side effects of radiation and chemotherapy include mucositis, xerostomia, nausea and emesis, which all contribute to decreased nutritional intake.^5^ Post-treatment sequela on the mandible and dentition can further play a role in impairing adequate nutritional intake.

Malnutrition and weight loss has been implicated as a prognostic factor following treatment for head and neck cancers. Poor nutritional status has been shown to triple the risk of death and is a stronger predictor of prognosis than tumour–node–metastasis stage.^6^ Weight loss prior to or during radiation therapy have both been independently associated with increased risk of death.^7^ Importantly, improving a patient's nutrition status reverses this trend and increases survival.^8,9^ In head and neck cancer, the role of enteral nutrition to reverse or prevent malnutrition is of significant interest because of high prevalence of dysphagia. The type of intervention (e.g. nasogastric *vs* gastric tubes) and the timing of intervention (e.g. prophylactic *vs* post-treatment) will often vary significantly based on institution.

Several guidelines have been developed to optimise and prioritise nutrition management in head and neck cancer patients to improve outcomes, but to date there has not been a systematic review of the quality and rigour in development of these guidelines. The Appraisal of Guidelines for Research and Evaluation II tool was established to assess the quality of current clinical practice guidelines, and its use has proven to be effective in a variety of fields, particularly otorhinolaryngology.^10^ The purpose of this study was to assess existing nutritional recommendations for head and neck cancer care to ensure rigour and clinical applicability using the Appraisal of Guidelines for Research and Evaluation II instrument.

## Materials and methods

The systematic review was conducted according to Preferred Reporting Items for Systematic Reviews and Meta-Analyses (‘PRISMA’) guidelines (see Table 1 in the supplementary material, available on *The Journal of Laryngology & Otology* website).^11^

### Literature search

A literature search was performed in the following three databases: Embase, Medline via PubMed and Scopus. The search terms included: [(‘nutrition’ OR ‘nutritional’) AND (‘head and neck cancer’) AND (‘guideline’ OR ‘consensus’ OR ‘recommendation’)]. Guidelines and consensus statements pertaining to management of nutrition in patients with head and neck cancer, regardless of treatment modality, were included in the analysis. Both national and international clinical practice guidelines were included; we excluded guidelines not available in English. For development groups that published multiple guidelines, the most recent guideline available was used.

### Data collection

General characteristics of the available guideline were evaluated during initial review and assessment for eligibility. Author and year of publication, developmental body, method of guideline development, relevant funding, region of origin, evidence used, guideline content and target users were abstracted and tabulated. Data for appraisal of each guideline were collected using a standardised form made available to individual reviewers, based on the six domains of quality and 23 individual items presented in the Appraisal of Guidelines for Research and Evaluation II instrument. These data were aggregated after independent appraisal and domain scores were calculated.

### Quality appraisal

Independent assessments of the selected clinical practice guidelines were performed by four authors (KP, BG, MS and TM) based on the Appraisal of Guidelines for Research and Evaluation II criteria. Prior to evaluation, all investigators completed the free, online training tool available on the Appraisal of Guidelines for Research and Evaluation website (www.agreetrust.org). The Appraisal of Guidelines for Research and Evaluation II instrument consists of 23 items assessing six quality domains: (1) scope and purpose, (2) stakeholder involvement, (3) rigour of development, (4) clarity of presentation, (5) applicability and (6) editorial independence. Each item was scored on a scale from 1 (strongly disagree) to 7 (strongly agree), where a score of 1 was given if the item was not addressed, and a score of 7 was recorded if the guideline fully addressed the item. Per the guidelines set forth by the Appraisal of Guidelines for Research and Evaluation II manual, the domains were scored according to the following formula:^12^




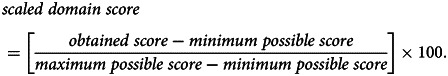




Overall scores for each guideline were calculated and reported as means. Protocol quality was rated as ‘high’ if 5 or more domains scored equal to or more than 60 per cent, ‘average’ if 3–4 domains scored equal to or more than 60 per cent, and ‘low’ if equal to or less than 2 domains scored equal to or more than 60 per cent.^13^

### Statistical analysis

In order to assess the interrater reliability among the four appraisers, an intraclass coefficient was calculated using RStudio integrated development environment software (Boston, USA). Intraclass coefficient was calculated as: poor (less than 0.20), fair (0.21–0.41), moderate (0.41–0.60), good (0.61–0.80) and very good (0.81–1.00) according to previous literature.^13^

## Results

**Fig. 1.**
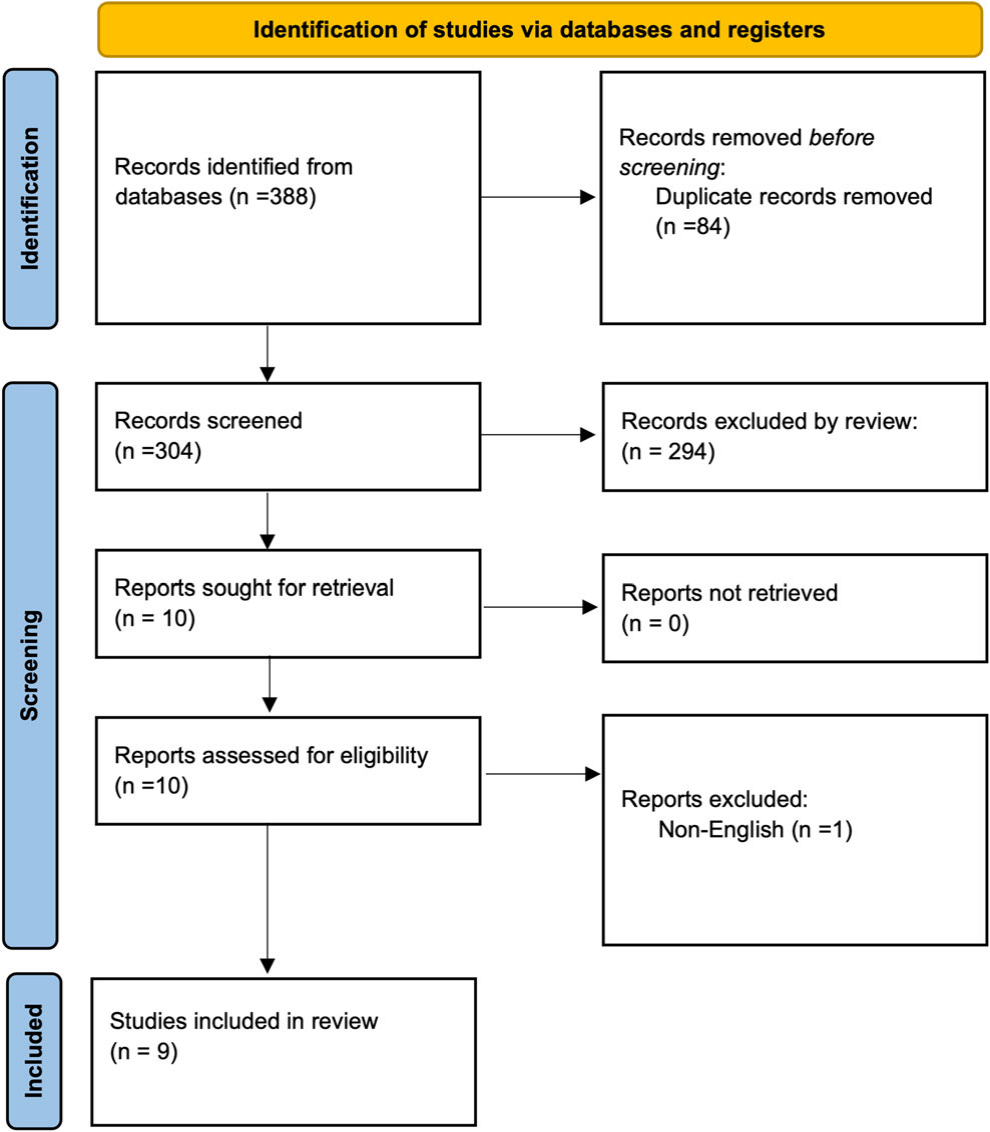
Flow diagram of the systematic literature search process.

The initial electronic search yielded 388 available reports. Duplicate records were removed, and a total of 304 articles were screened by title and abstract for exclusion and inclusion criteria. From the initial screen, 10 were selected for a full review. After review of the full text, nine guidelines were determined to meet inclusion criteria and selected for evaluation (Figure 1).

### Guideline characteristics

A summary of the general characteristics for each clinical practice guideline is provided in Table 1. The nine guidelines evaluated represented five different countries. The USA had the most, with four guidelines. Eight of the guidelines focused on nutritional management in all head and neck cancers regardless of treatment modality, whereas the guideline by Lin *et al*.^14^ evaluated nutrition management in head and neck cancer patients specifically undergoing chemoradiation. Five of the guidelines were developed by multidisciplinary national committees, including the Clinical Oncological Society of Australia,^15^ National Comprehensive Cancer Network,^16^ National Institute for Health and Care Excellence (NICE),^17^ UK National Multidisciplinary Guidelines^18^ and the Taiwan Head and Neck Oncology Society.^14^ The other guidelines included peer-reviewed articles (Wagner^19^ and Gill *et al*.^20^) and book chapters (Sarangapani *et al*.^21^ and Ackerman *et al*.^22^).

**Table 1.** Guideline characteristics

Author & year	Guideline developer(s)	Region of origin	Funding	Evidence base	Target population
Ackerman *et al*.^22^ (2018)	City of Hope National Medical Center & Cedars-Sinai Medical Center	USA	Not available	Author experience	All H&N cancer
Findlay *et al*.^27^ (2011)	Clinical Oncological Society of Australia	Australia	Cancer Institute NSW Oncology Group (H&N)	Systematic literature review, expert consensus	All H&N cancer
Gill *et al*.^20^ (2018)	UC Davis School of Medicine	USA	Not available	Author experience	All H&N cancer
Lin *et al.*^14^ (2018)	National Taiwan University Hospital	Japan	Abbott Nutrition	Systematic literature review, expert consensus	H&N cancer undergoing chemoradiation
Pfister *et al.*^16^ (2021)	National Comprehensive Cancer Network	USA	NCCN Foundation	Systematic literature review, expert consensus	All H&N cancer
NICE (2007)^17^	National Institute for Health and Care Excellence	UK	NICE	Systematic literature review, expert consensus	All H&N cancer
Sarangapani *et al.*^21^ (2013)	University of Illinois at Chicago	USA	Not available	Author experience	All H&N cancer
Talwar *et al.*^18^ (2016)	UK National Multidisciplinary Guidelines	UK	National Health Service	Expert consensus	All H&N cancer
Wagner^19^ (2020)	Medical University of Vienna	Austria	Medical University of Vienna	Systematic literature review	All H&N cancer

H&N = Head and Neck; NSW = New South Wales; UC = University of California; NCCN = National Comprehensive Cancer Network; NICE = The National Institute for Health and Care Excellence

### Quality appraisal

After independent appraisal of all clinical practice guidelines by our four reviewers, scaled domain scores were calculated for the six quality domains of the Appraisal of Guidelines for Research and Evaluation II instrument (Table 2). Per our reviewers’ appraisals, there was significant variability seen in the quality among the nine clinical practice guidelines reviewed based on these scaled domain scores. The scores ranged from 0 per cent (domain 6 for Sarangapani *et al*.^21^ and Ackerman *et al*.^22^) to 100 per cent (domain 1, 2 and 6 for NICE and domain 6 for Lin *et al*.^14^). The ‘scope and purpose’ domain had the lowest variability as well as the highest mean domain score (75.5 ± 0.17). Both the NICE and Clinical Oncological Society of Australia guidelines achieved overall ratings of ‘high’, with each having all 6 domains at more than 60 per cent. The Taiwan Head and Neck Oncology Society and National Comprehensive Cancer Network guidelines received ‘average’ ratings, and the remaining five guidelines scored ‘low’.

**Table 2.** Guideline domain scores and overall quality

Guideline	Domain 1	Domain 2	Domain 3	Domain 4	Domain 5	Domain 6	Overall mean (score)	Overall quality
	Scope and purpose (score)	Stakeholder involvement (score)	Rigour of development (score)	Clarity and presentation (score)	Applicability (score)	Editorial independence (score)		
Sarangapani *et al.*,^21^ 2013 (%)	48.6	15.3	15.1	45.8	11.5	0.0	22.7	Low
Taiwan 2018 (%)^14^	94.4	59.7	69.8	97.2	30.2	100.0	75.2	Average
NICE 2007 (%)^17^	100.0	100.0	91.7	97.2	71.9	100.0	93.5	High
Talwar *et al.*,^18^ 2016 (%)	72.2	33.3	16.7	95.8	52.1	4.2	45.7	Low
Ackerman *et al.*,^22^ 2018 (%)	70.8	27.8	16.7	41.7	26.0	0.0	30.5	Low
NCCN 2021 (%)^16^	69.4	62.5	56.8	76.4	30.2	75.0	61.7	Average
Gill *et al.*,^20^ 2018 (%)	69.4	11.1	22.4	68.1	20.8	45.8	39.6	Low
Wagner^19^ 2020 (%)	55.6	8.3	14.6	47.2	15.6	64.6	34.3	Low
COSA 2011 (%)^15^	98.6	97.2	94.8	100.0	80.2	100.0	95.1	High
Mean ± SD	75.5 ± 17	46.1 ± 33	44.3 ± 032	74.4 ± 023	37.6 ± 23	54.4 ± 41		

Taiwan = Taiwan Head and Neck Oncology Society; NICE = National Institute for Health and Care Excellence; NCCN = National Comprehensive Cancer Network; COSA = Clinical Oncological Society of Australia; SD = standard deviation

### Intraclass correlation coefficient for interrater reliability

Following scaled domain score calculations, intraclass correlation coefficients were utilised to assess reliability between raters for each domain, which have been summarised in Table 3. In five of the six Appraisal of Guidelines for Research and Evaluation II domains (domains 1, 2, 3, 5 and 6), our four independent reviewers achieved intraclass coefficient scores of more than 0.81, suggesting very good interrater reliability and almost complete agreement regarding the quality of each clinical practice guideline in that domain. In the remaining domain, clarity of presentation, our reviewers achieved an intraclass coefficient score of 0.78, which suggests good interrater reliability (more than 0.61) and a strong level of agreement regarding domain score for each clinical practice guideline. No intraclass coefficient was calculated that fell below the threshold of good intraclass coefficient suggesting strong agreement, indicating sufficient correlation between reviewers in guideline appraisal.

**Table 3.** Intraclass correlation coefficients across all domains

Appraisal of Guidelines for Research and Evaluation II domain	Intraclass correlation coefficient	95% confidence interval
Scope and purpose	0.87	0.77 to 0.93
Stakeholder involvement	0.95	0.92 to 0.98
Rigour of development	0.91	0.87 to 0.94
Clarity of presentation	0.78	0.6 to 0.89
Applicability	0.82	0.69 to 0.9
Editorial independence	0.90	0.79 to 0.96

## Discussion

Head and neck cancer relies on a variety of efforts to reduce complications during treatment.^23^ Nutritional status, both pre-treatment and post-treatment, has been found to be a key predictor of patient outcomes.^7,24^ Malnutrition is particularly common in head and neck cancers because of high prevalence of co-morbid conditions such as alcohol use and the direct impact of the tumour burden on the upper aerodigestive system. Treatment modalities for head and neck cancer can further induce difficulties via surgical disruption of normal anatomical structures and through toxicities related to adjuvant therapies.^25,26^ In recognition of the importance that nutrition plays on outcomes, clinical practice guidelines have been developed to provide standardised, evidence-backed interventions to optimise nutritional status throughout all stages of treatment for head and neck cancer. The present study is the first to assess the quality and consistency of clinical practice guidelines for nutritional interventions in patients with head and neck cancer. We identified nine guidelines and consensus statements from across the world and demonstrated that specific components should be optimised and revamped to increase the completeness and transparency of these guidelines.

The analysis showed both the ‘scope and purpose’ and ‘clarity and presentation’ domains were rated as high quality across clinical practice guidelines, achieving mean scores of 75.5 per cent and 75.4 per cent, respectively. The highest rated guidelines tended to explicitly identify populations that would benefit from nutritional interventions. They included specific recommendations regarding the timing and type of nutritional support for head and neck cancer patients. The lowest rated guidelines often discussed nutrition management in broader terms without first identifying a question or objective. Most guidelines achieved high scores in ‘clarity of presentation’, with five receiving scores of more than 90 per cent, representing how recommendations were explicitly laid out with little ambiguity.

The ‘stake holder involvement’ domain refers to the involvement of professionals and target populations in the development of guidelines. Only two guidelines achieved satisfactory performance in this domain. Nutrition management in head and neck cancer is a complex process that requires a multidisciplinary team of doctors, dieticians, speech language pathologists, clinical nutritionists and pharmacists. For example, speech language pathologists and dieticians play vital roles in identifying at risk patients and providing swallowing strategies and therapy during and after treatment. In addition, clinical nutritionists aid in optimising nutritional intake to maximise interventions. Three guidelines (NICE, National Comprehensive Cancer Network and Clinical Oncological Society of Australia) sought feedback and input from these groups during development, and only NICE and Clinical Oncological Society of Australia achieved overall high-quality ratings.

The ‘applicability’ domain is especially important to demonstrate that recommendations can be feasibly implemented. Unfortunately, this domain received the lowest mean scaled score of 37.6 per cent. Seven guidelines scored less than 60 per cent and failed to adequately describe barriers to implementation, associated costs and methods for continued auditing of guidelines in the future. The Clinical Oncological Society of Australia guideline was the sole guideline to achieve exceptional performance with a scaled score of more than 80 per cent. Uniquely, the Clinical Oncological Society of Australia has demonstrated commitment to auditing their initial guidelines and publishing analysis of adherence and outcomes of their published guidelines.^27–29^

‘Rigour of development’ and ‘editorial independence’ have been shown to have the strongest influence on overall guideline quality and recommendation for use.^30^ ‘Rigour of development’ encompasses the methodology used in developing the guideline as well as the review by external experts. ‘Editorial independence’ looks at funding and conflict of interests. Both domains received average mean scores of 44.3 per cent and 54.45 per cent, respectively. Only three guidelines (by Lin *et al*., NICE and Clinical Oncological Society of Australia), all developed by national multidisciplinary groups, were rated as high quality in ‘rigour of development’. The four peer-reviewed articles and book chapters all received scores less than 20 per cent; the biggest flaws tended to be a weak systematic search methodology, lack of a method to obtain consensus recommendations and no means of continued updating of recommendations. The Taiwan, NICE and Clinical Oncological Society of Australia guidelines scored remarkably well in ‘editorial independence’, achieving scores of 100 per cent. Each guideline demonstrated unbiased reporting with explicit disclosure of conflicts and funding. Three guidelines (Sarangapani *et al*.,^21^ UK National Multidisciplinary Guidelines and Ackerman *et al*.^22^) scored very poorly: less than 5 per cent with no mention of possible sources of bias.

Only the NICE and Clinical Oncological Society of Australia guidelines achieved a high-quality rating with six domains scoring more than 60 per cent and agreement between all reviewers to recommend the guidelines for use. Both organisations publish a variety of guidelines for different specialties and follow similar systematic methodologies in the creation of each, potentially explaining the high quality of their guidelines on nutrition in head and neck cancer. Interestingly, all four peer-reviewed articles and book chapters scored low on overall quality, begging the question if the support of a large national organisation is needed to develop high-quality clinical practice guidelines.

### Recommendations

Utilising the quality threshold set forth by the Appraisal of Guidelines for Research and Evaluation II instruments as a framework, recommendations for the nutritional management of head and neck cancer patients can be summarised as follows. (1) Early identification and intervention are essential in improving nutritional status in patients with head and neck cancer. (2) Nutritional intervention with a multidisciplinary team that includes a dietitian throughout the treatment course is paramount. (3) Patients should undergo pre-treatment nutrition screening at the time of diagnosis. Patients undergoing radiation or who are otherwise identified as high risk for malnutrition should be referred to a dietician for early intervention. (4) Use of objective screening tools such as the Malnutrition Screening Tool^31^ and Patient-Generated Subjective Global Assessment^32^ should be employed for nutrition screening. (5) Prophylactic feeding tube should be considered in patients who are high risk for malnutrition based on nutrition status, tumour site and stage, treatment modality, presence of pre-treatment dysphagia, and social support.

### Limitations

The study has several limitations in evaluating the quality of each guideline. The Appraisal of Guidelines for Research and Evaluation II tool assesses the quality and objectivity of the creation of the guidelines, which often, though not always, correlates to the validity of the recommendations. The reviewers did not, however, analyse and compare recommendations between each guideline. Since many guidelines are produced by international groups, it is possible that guidelines published in languages other than English were not included in our analysis. Although we achieved good intraclass coefficient in all domains, scoring of each domain is subjective to each reviewer and makes comparisons to other studies using the Appraisal of Guidelines for Research and Evaluation II tool difficult.

## Conclusion

High-quality clinical practice guidelines and recommendations based on multidisciplinary and rigorous unbiased methodological development can create pathways for providers to optimise outcomes for patients. A variety of guidelines have been developed to address the nutritional status in patients with head and neck cancer. Using the Appraisal of Guidelines for Research and Evaluation II tool, the overall quality of these clinical practice guidelines and consensus statements was evaluated. Out of nine guidelines identified, only the NICE and Clinical Oncological Society of Australia guidelines were rated as high quality and recommended for use in clinical practice. There remains opportunity to improve the quality of published guidelines, particularly regarding applicability and the rigour of development.

## Supplementary material

For supplementary material accompanying this paper visit https://doi.org/10.1017/S002221512200055X. click here to view supplementary material
